# Supplementary material for: Acute and Chronic Systemic Inflammation: Features and Differences in the Pathogenesis, and Integral Criteria for Verification and Differentiation
Source: Int J Mol Sci. 2023 Jan 6;24(2):1144. doi: 10.3390/ijms24021144 (PMC9862412; doi:10.3390/ijms24021144)
Supplement: Supplementary file 1 [file ijms-24-01144-s001.zip › ijms-2034730-SI.pdf]

Supplementary Materials

Table S1. Descriptive statistics of markers in the groups of the acute nosologies.

Groups	IL-6, pg / ml	IL-8, pg / ml	IL-10, pg / ml	TNFα, pg / ml	CRP, mg / dl	RL – Reactivity Level	SI score
Control 1 (donors), n=50	2.02±0.45	5.58±1.56	4.90±0	4.33±1.03	0.26±0.24	0.00±0.00	0
	1.90 / 1.90÷4.60	4.90 / 4.90÷5.10	4.90 / 4.90÷4.90	3.90/ 3.90÷4.20	0.20/ 0.10÷0.30	0.00/0.0÷0.0	0
Control 1 (donors), n=50	2.32±1.19	9.70±5.89	4.90±0	7.59±12.92	0.52±0.53	0.18±0.39	0
	1.90 / 1.90÷1.90	7.60 / 5.60÷10.90	4.90 / 4.90÷4.90	4.25 / 3.90÷6.10	0.35/ 0.20÷0.70	0.00/0.0÷0.00	0
Multiple injuries on days 1-2, n=51	322.14±540.18	122.83±224.66	198.98±488.92	23.84±44.54	83.22±165.51	3.10 ±0.90	5.80±1.75
	111.00 / 51.30÷247.00	52.50 / 20.30÷100.00	52.20 / 6.00÷ 187.00	8.50 / 4.40÷17.60	37.00 / 4.00÷87.00	3.00/ 2.00÷4.00	6.00/5.00÷7.00
Multiple injuries on days 5-7, n=18	810.54±1751.68	170.76±429.19	31.72±50.39	16.06±11.51	254.89±244.73	2.72 ±0.96	5.17±1.89
	61.95/ 34.00÷902.00	28.10/ 12.70÷55.60	5.00/ 4.90÷28.40	13.95/ 5.40÷22.60	174.00/ 98.00÷280.00	2.50 / 2.00÷4.00	5.00 / 3.00÷7.00
Sepsis non-resuscitative on days 1-2, n=40	48.68±91.18	18.62±13.79	8.69±9.21	16.17±8.26	11.60±15.69	1.90±0.67	3.03±0.86
	13.35 / 4.85÷31.25	14.15 / 10.85÷24.80	<5	14.25 / 10.30÷21.05	5.80 / 1.80÷16.00	2.00 / 1.00÷2 00	3.00 / 2.00÷3 00
Sepsis non-resuscitative on days 1-2, n=46	1680.48÷3776.95	830.78÷1998.67	92.22÷202.75	248.96÷693.51	28.64÷30.57	3.37÷0.99	5.59÷1.80
	204.5 / 56.90÷507.00	104.90 / 21.20÷361.0	16.70 / <5÷102.00	17.60 / 7.80÷51.10	19.35 / 10.70÷36.20	3.00 / 3.00÷4.00	6.00 / 4.00÷6.00
Sepsis on days 5-7, n=13	322.35÷510.42	478.34÷1081.30	28.71÷46.43	153.21÷338.58	19.62÷13.04	3.38÷0.65	5.92÷0.95
	71.90 / 47.20÷324.00	102.00 / 29.30÷312.00	13.40 / 5.20÷31.70	26.70 / 19.40÷100.00	18.50 / 12.50÷23.50	3.0 / 3.00÷4.00	6.00 / 5.00÷6.00
Septic shock, n=14	4168.86±5289.08	698.57± 690.14	116.51± 95.40	258.28±616.42	25.34±16.44	4.14±0.77	7.00±1.11
	2583.50 / 341.00÷4074.00	493.00 / 149.00÷ 999.00	93.20 / 47.60÷176.00	45.50 / 26.00÷141.00	28.25 / 10.80÷34.00	4.00 / 4.00÷5.00	7.00 / 6.00÷8 00
Tertiary sepsis MODS, n=34	254.58÷446.88	500.85÷1142.25	16.01÷23.88	67.45÷100.88	24.53÷20.63	3.09±0.67	5.35±1.01
	58.70 / 35.20÷143.00	58.70 / 35.20÷143.00	5.85 / 4.90÷18.00	24.60 / 14.70÷65.60	21.50 / 8.00÷37.30	3.00 / 3.00÷3.00	5.00 / 5.00÷6.00
Septic shock tertiary sepsis, n=17	410.06± 1376.55	140.46± 157.23	11.25±10.35	17.99±7.01	20.62±20.57	2.71±0.59	5.65±0.70
	75.10 / 59.60÷99.00	65.90 / 48.10÷185.00	7.80 / 4.90÷11.90	17.80 / 15.50÷20.00	11.10 / 8.50÷25.10	3.00 / 2.00÷3.00	6.00 / 5.00÷6.00
Lethal outcomes n=71	1636.64±3357.43	671.47± 1635.00	157.92± 432.63	99.47±319.90	21.81±20.53	3.55±0.82	6.38±1.28
	235.00 / 69.70÷1465.00	111.00 / 62.80÷410.00	23.00 / 8.00÷176.00	20.00 / 12.90÷36.20	18.20 / 6.80÷29.70	4.00/ 3.00÷4.00	6.00/ 5.00÷7.00

Note. The descriptive statistics are presented by their main characteristics: m±SD and Me / 25%÷75% – quartiles; m – mean value, Me – median, SD – standard deviation. Data <0.2 or <0.5 is below the sensitivity of the diagnostic kit.

Table S1. Descriptive statistics of markers in the groups of the acute nosologies (continued).

Groups	Troponin I, pg / ml	Myoglobin, pg / ml	Cortisol, nmole / l	SOFA score
**Control 1 (donors), n=50**	<0.2	13.56 ± 4.22	348.86 ± 129.28	0
	<0.2	13.00 / 10.70 ÷ 15.20	321.50 / 262.00 ÷ 426.00	0
**Control 1 (donors), n=50**	<0.2	24.13±6.61	415.14 ± 121.80	0
	<0.2	23.55 / 19.60÷26.30	380.50 / 328.00 ÷ 490.00	0
**Multiple injuries on days 1-2, n=51**	0.67 ± 1.46	496.80 ± 304.35	1088.53 ± 692.35	4.96 ± 1.87
	<0.2 / <0.2 ÷0.35	524.00 / 231.00 ÷801.00	974.00 / 582.00 ÷ 1434.00	5.00 / 4.00 ÷ 6.00
**Multiple injuries on days 5-7, n=18**	0.56±0.80	239.24 ±235.23	1261.18±1146.33	6.75 ± 4.25
	0.215/ 0.19÷0.42	162.00/ 63.50÷291.00	828.00 / 529.00 ÷2130.00	5.00 / 5.00 ÷8.50
**Sepsis non-resuscitative on days 1-2, n=40**	0.56±2.33	117.90±250.77	500.85±283.20	3.20±1.14
	<0.2	50.70 / 29.25÷104.40	407.50 / 270.50÷703.00	3.00 / 2.00÷4 00
**Sepsis non-resuscitative on days 1-2, n=46**	2.54÷14.85	197.52÷226.04	1187.39÷684.73	5.50÷2.30
	0.19 / 0.19÷0.22	108.00 / 53.40÷205.00	916.00 / 652.00÷1520.00	5.00 / 3.00÷7.00
**Sepsis on days 5-7, n=13**	0.85÷1.35	238.98÷260.90	1073.38÷831.28	5.77÷2.20
	0.19 / 0.19÷1.10	120.00 / 54.20÷419.00	853.00 / 737.00÷1049.00	6.00 / 4.00÷7.00
**Septic shock, n=14**	0.76±1.86	224.47± 268.33	1467.29 ±749.64	9.75±2.22
	0.21 / 0.19÷0.27	121.00 / 31.00÷289.00	1430.00 / 970.00÷1920.00	10.00 / 8.50÷11.00
**Tertiary sepsis MODS, n=34**	0.28±0.29	184.43± 216.52	633.53± 369.18	4.55±2.72
	0.19 / 0.19÷0.24	106.00 / 64.80÷206.00	582.50 / 356.00÷840.00	4.00 / 3.00÷5.00
**Septic shock tertiary sepsis, n=17**	1.58±2.83	276.78± 297.44	1161.12±870.01	8.06±3.23
	0.23 /0.19÷1.10	166.00 / 105.00÷ 259.00	1015.00 / 513.00÷1396.00	8.00 / 5.00÷11.00
**Lethal outcomes n=71**	2.29±12.01	359.27± 329.80	1255.71± 978.20	7.99 ± 3.55
	0.23 / 0.19÷0.61	206.00 / 88.40÷640.00	974.00 / 520.00÷ 1787.00	8.00 / 5.00÷10.00

Note. The descriptive statistics are presented by their main characteristics: m±SD and Me / 25%÷75% – quartiles; m – mean value, Me – median, SD – standard deviation. Data <0.2 or <0.5 is below the sensitivity of the diagnostic kit.

Table S2. Descriptive statistics of markers in the groups of the chronic nosologies.

Groups	IL-6, pg / ml	IL-8, pg / ml	TNFα, pg / ml	CRP, mg / dl	RL – Reactivity Level	ChSI score
Control 1 (donors), n=50	2.02±0.45	5.58±1.56	4.33±1.03	0.26±0.24	0.00±0.00	0
	1.90 / 1.90÷4.60	4.90 / 4.90÷5.10	3.90/ 3.90÷4.20	0.20/ 0.10÷0.30	0.00/0.0÷0.0	0
Control 1 (donors), n=50	2.32±1.19	9.70±5.89	7.59±12.92	0.52±0.53	0.18±0.39	0.18±0.39
	1.90 / 1.90÷1.90	7.60 / 5.60÷10.90	4.25 / 3.90÷6.10	0.35/ 0.20÷0.70	0.00/0.0÷0.00	0.00/0.00÷0.00
SLE, n=49	548.63±1929.33	2097.18±5224.66	159.26±280.39	0.70±0.99	2.94 ±1.27	3.69±1.58
	58.70 / 8.80÷165.00	588.00 / 131.00÷1942.00	69.50 / 36.20÷144.00	0.35 / 0.13÷0.85	3.00/ 2.00÷4.00	4.00/3.00÷5.00
RA, n=42	14.61±18.22	25.09±48.58	12.17±14.99	2.07±2.65	0.93 ±0.78	1.83±1.25
	4.90/ 1.90÷20.60	6.85/ 4.90÷10.20	5.65/ 3.90÷14.80	1.80/ 0.33÷2.42	1.00 / 0.00÷1.00	2.00 / 1.00÷3.00
ReA, n=30	7.54±11.65	33.80±71.66	11.97±11.86	1.54±2.00	0.73 ±0.79	1.40±1.22
	2.05/ 1.90÷5.60	4.95/ 4.90÷11.80	8.00/ 4.80÷11.20	0.47/ 0.12÷2.22	1.00 / 0.00÷1.00	1.00 / 0.00÷2.00
AS, n=27	5.47±5.46	16.86±27.39	13.77±11.96	1.82±1.96	0.78 ±0.80	1.00±1.04
	4.00/ 1.90÷7.30	5.60/ 4.90÷9.40	10.30/ 8.10÷12.80	0.99/ 0.55÷2.84	1.00 / 0.00÷1.00	1.00 / 0.00÷2.00
PsA, n=12	8.88±7.55	15.04±31.83	6.28±4.92	1.64±1.58	0.83 ±0.72	1.08±1.00
	5.60/ 4.40÷11.40	4.90/ 4.90÷7.75	3.90/ 3.90÷5.20	1.23/ 0.26÷2.86	1.00 / 0.00÷1.00	1.00 / 0.00÷2.00
RHD, n=15	3.29±1.57	40.01±91.56	13.71±14.12	0.56±0.61	0.60 ±0.74	0.93±1.03
	2.60/ 1.90÷4.20	6.20/ 4.90÷27.10	4.90/ 3.90÷27.20	0.35/ 0.19÷0.75	0.00 / 0.00÷1.00	1.00 / 0.00÷1.00
CHF, n=49	6.61±7.08	16.27±48.25	7.36±4.37	1.49±2.41	0.57 ±0.68	0.82±0.91
	4.10/ 2.60÷7.30	6.50/ 4.90÷10.20	6.00/ 3.90÷8.90	0.65/ 0.30÷1.22	0.00 / 0.00÷1.00	1.00 / 0.00÷1.00
ESRD, n=42	8.52±11.48	233.00±377.57	100.66±117.67	0.86±1.04	2.00 ±0.83	3.43±106
	5.50/ 3.00÷7.80	162.50/ 27.80÷289.00	58.85/ 24.90÷138.00	0.44/ 0.18÷1.32	2.00 / 2.00÷2.00	4.00 / 3.00÷4.00
CLI, n=38	16.89±17.29	49.00±57.86	13.23±12.18	5.91±4.68	1.68 ±0.74	3.00±1.56
	11.20/ 3.90÷22.10	18.90/ 12.00÷66.30	8.90/ 3.90÷15.50	4.69/ 1.79÷10.40	2.00 / 1.00÷2.00	3.00 / 2.00÷4.00
CNW, n=42	3.56±1.93	11.54±9.68	12.63±3.06	1.01±1.29	0.83 ±0.44	1.40±0.86
	2.85 / 1.90÷5.10	7.70 / 5.70÷13.40	12.90 /10.60÷14.70	0.74 / 0.07÷1.29	1.00/ 1.00÷1.00	1.00/ 1.00÷2.00
AIT, n=29	2.99±2.24	6.52±4.01	5.86±2.74	0.43±0.62	0.21±0.41	0.24±0.51
	2.00 / 1.90÷2.70	4.90 / 4.90÷5.70	5.30 / 3.90÷7.10	0.23 / 0.07÷0.47	0.00 / 0.00÷0.00	0.00 / 0.00÷0.00
PID, n=16	5.48±1.92	7.56±8.45	5.81±4.10	0.72±0.61	0.25±0.45	0.25±0.45
	4.90 / 4.90÷4.90	4.90 / 4.90÷6.20	3.90 / 3.90÷5.75	0.47 / 0.24÷1.29	0.00 / 0.00÷0.50	0.00 / 0.00÷0.50
Menopausal syndrome, n=16	1.91±0.03	8.34±3.59	5.00±2.04	0.38±0.50	0.06±0.25	0.06±0.25
	1.90 / 1.90÷1.90	7.00 / 4.90÷11.70	3.90 / 3.90÷5.90	0.18 / 0.08÷0.52	0.00 / 0.00÷0.00	0.00 / 0.00÷0.00
CRAD, n=23	2.80±1.50	56.03±126.15	38.78±41.00	0.90±2.02	1.17±0.65	2.26±1.29
	1.90 / 1.90÷3.10	6.90 / 4.90÷10.30	27.30 /16.70÷36.80	0.30 / 0.04÷0.75	1.00 / 1.00÷1.00	2.00 / 1.00÷3.00
Normal function of renal allograft, n=24	2.51±1.43	9.94±9.81	14.59±11.57	0.57±1.10	0.58±0.78	0.92±0.83
	1.90 / 1.90÷1.90	4.90 / 4.90÷10.25	10.80 / 7.85÷15.60	0.07 / 0.03÷0.36	0.00 / 0.00÷1.00	1.00 / 0.00÷2.00
PAPS, n=5	33.80±32.19	634.00±486.16	35.38±19.47	0.97±0.55	2.80±0.45	3.80±0.45
	15.80 / 15.70÷31.90	541.0 / 267.0÷653.0	27.60 /20.40÷55.80	0.75 / 0.73÷1.13	3.00 / 3.00÷3.00	4.00 / 4.00÷4.00

Note. The descriptive statistics are presented by their main characteristics: m±SD and Me / 25%÷75% – quartiles; m – mean value, Me – median, SD – standard deviation.

Table S2. Descriptive statistics of markers in the groups of the chronic nosologies (continued).

Groups	Myoglobin, pg / ml	Cortisol, nmole / l
Control 1 (donors),	13.56 ± 4.22	348.86 ± 129.28
n=50	13.00 / 10.70 ÷ 15.20	321.50 / 262.00 ÷ 426.00
Control 1 (donors),	13.56 ± 4.22	348.86 ± 129.28
n=50	13.00 / 10.70 ÷ 15.20	321.50 / 262.00 ÷ 426.00
SLE, n=49	18.34 ± 13.61	179.15 ± 126.48
	12.60 / 10.10 ÷21.50	123.00 / 95.00 ÷ 227.00
RA, n=42	17.12 ±8.43	423.05±264.12
	15.10/ 11.70÷19.50	434.50 / 224.00 ÷603.00
ReA, n=30	18.27 ±8.21	460.84 ±303.95
	16.50/ 12.20÷21.90	449.50/ 200.00÷724.00
AS, n=27	23.68 ±17.15	354.67 ±177.78
	14.80/ 11.60÷37.10	308.00/ 198.00÷495.00
PsA, n=12	23.83 ±16.62	392.33 ±168.56
	18.40/ 16.00÷25.65	392.50/ 233.00÷474.50
RHD, n=15	28.09±15.18	556.64 ±348.90
	20.30 / 19.10÷44.30	400.00 / 325.00÷676.00
CHF, n=49	25.85 ±9.76	449.77 ±95.05
	23.30/ 19.00÷30.20	450.00/ 381.00÷491.00
ESRD, n=42	135.19 ±66.41	537.19 ±166.79
	113.00/ 84.10÷181.00	535.00/ 427.00÷633.00
CLI, n=38	83.06 ±91.42	627.21 ±178.55
	45.50/ 29.00÷119.00	611.50/ 521.00÷757.00
CNW, n=42	72.21 ±53.37	660.45 ±287.51
	63.05/ 27.30÷89.70	619.50/ 450.00÷853.00
AIT, n=29	16.10±4.67	363.48±117.80
	15.20/ 12.50÷19.40	352.00 / 302.00÷403.00
PID, n=16	We did not	We did not
Menopausal syndrome,	19.94±8.80	321.44±98.01
n=16	17.30 / 14.80÷20.25	279.50 / 246.00÷404.00
CRAD, n=23	55.52±48.41	208.81±302.73
	43.30 / 32.00÷65.00	113.00 / 74.50÷259.00
Normal function of	28.80±11.14	224.15±161.34
renal allograft, n=24	28.20 / 19.20÷36.45	214.00 / 85.25÷320.50
PAPS, n=5	We did not	We did not

Note. The descriptive statistics are presented by their main characteristics: m±SD and Me / 25%÷75% – quartiles; m – mean value, Me – median, SD – standard deviation.

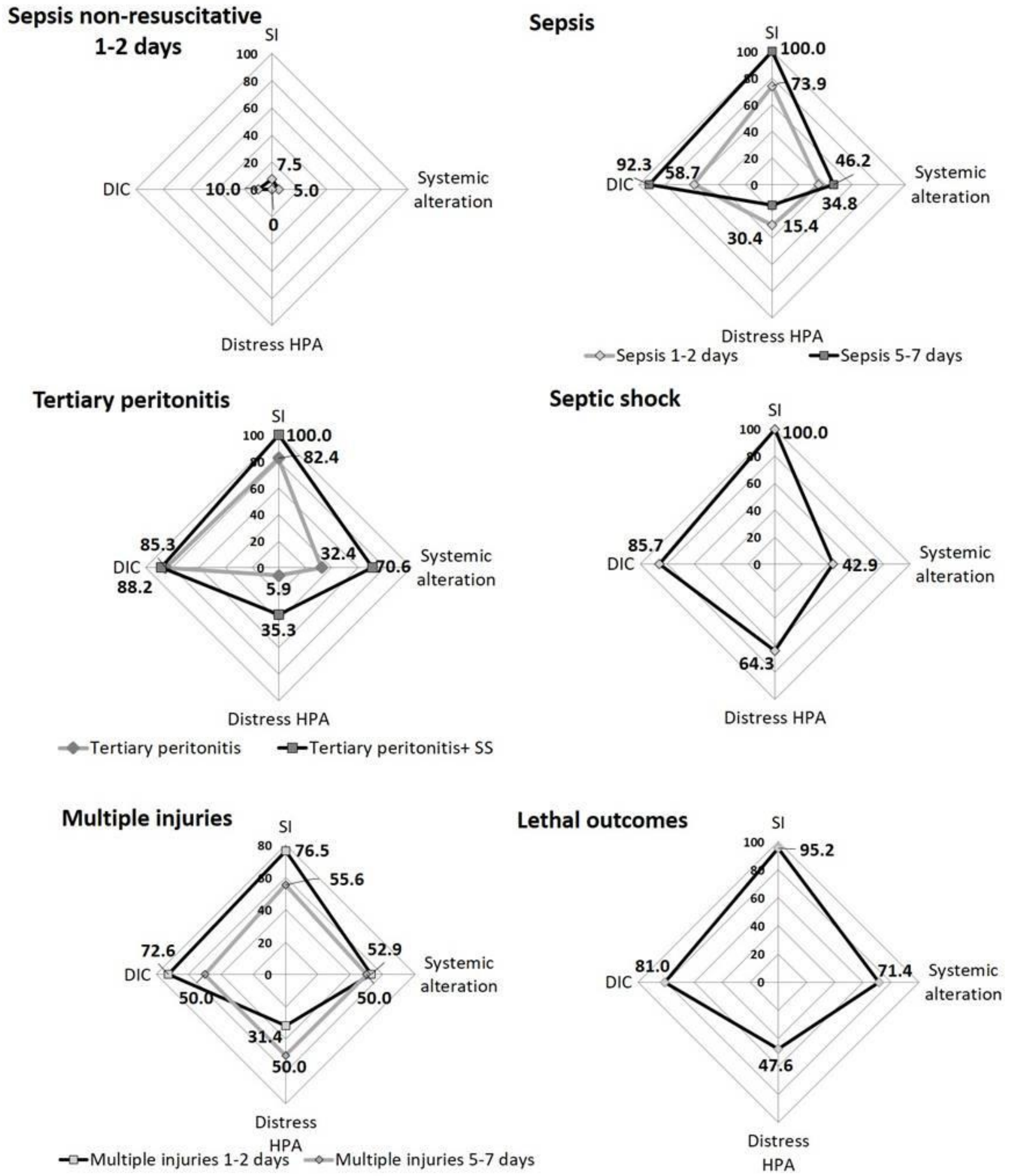

Figure S1. The phenomenological structure of the SI process-complex in the groups of the acute nosologies.

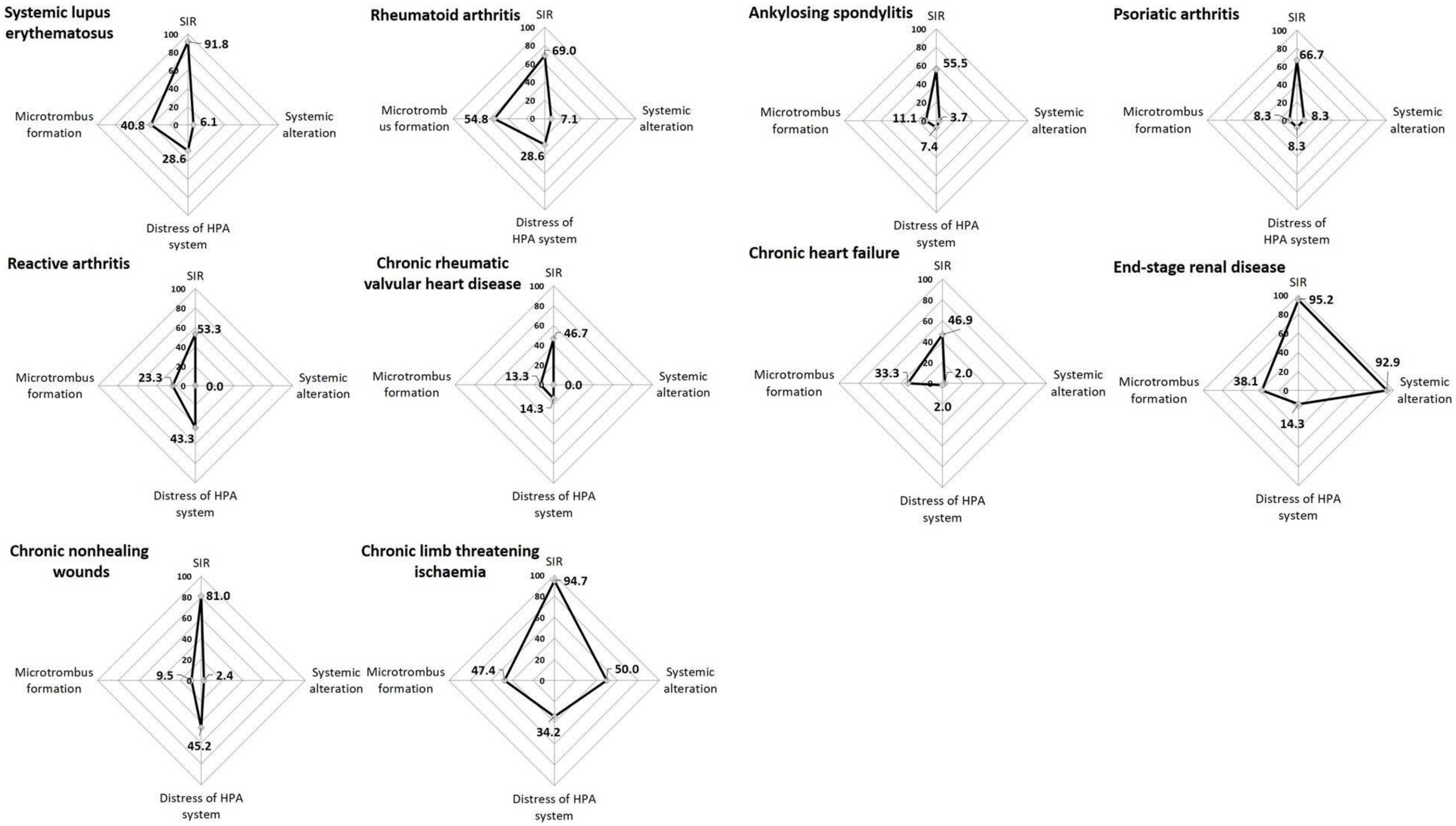

Figure S2. The phenomenological structure of the ChSI process-complex in the groups of the chronic nosologies. 1
